# Response to Toshihide Tsuda, Yumiko Miyano and Eiji Yamamoto [1]

**DOI:** 10.1186/s12940-022-00913-4

**Published:** 2022-10-26

**Authors:** Colin L. Soskolne, Lisa A. Bero, Shira Kramer, Michael Gochfeld, Juan Pablo Ramos-Bonilla, Jennifer Sass, Carl F. Cranor, Shailesh Advani, Daniele Mandrioli

**Affiliations:** 1 School of Public Health, University of Alberta, Edmonton, AB Canada; 2 Center for Bioethics and Humanities, University of Colorado Anschutz Medical Campus, Aurora, CO USA; 3 Epidemiology International, Hunt Valley, MD USA; 4 Environmental and Occupational Health Sciences Institute, Rutgers Biomedical and Health Sciences, Piscataway, NJ USA; 5 Department of Civil and Environmental Engineering, Universidad de Los Andes, Bogotá, Colombia; 6 Natural Resources Defense Council, Washington, DC USA; 7 George Washington University, Washington, DC USA; 8 Departments of Philosophy and Environmental Toxicology, University of California, Riverside, CA USA; 9 Terasaki Institute of Biomedical Innovation, Los Angeles, CA USA; 10 Georgetown University School of Medicine, Washington, DC USA; 11 Cesare Maltoni Cancer Research Centre, Ramazzini Institute, Bologna, Italy

**Keywords:** Biological sciences, Epidemiological methods, Ethics guidelines, Evaluation, Forensic epidemiology, Misuse, Risk of bias, Systematic framework, Transparency, Toolkit

## Abstract

**Background:**

In August 2021, we published in *Environmental Health* a Toolkit for detecting misused epidemiological methods with the goal of providing an organizational framework for transparently evaluating epidemiological studies, a body of evidence, and resultant conclusions. Tsuda et al., the first group to utilize the Toolkit in a systematic fashion, have offered suggestions for its modification.

**Main body:**

Among the suggested modifications made by Tsuda et al., we agree that rearrangement of Part A of the Toolkit to reflect the sequence of the epidemiological study process would facilitate its usefulness. Expansion or adaptation of the Toolkit to other disciplines would be valuable but would require the input of discipline-specific expertise. We caution against using the sections of the Toolkit to produce a tally or cumulative score, because none of the items are weighted as to importance or impact. Rather, we suggest a visual representation of how a study meets the Toolkit items, such as the heat maps used to present risk of bias criteria for studies included in Cochrane reviews. We suggest that the Toolkit be incorporated in the sub-specialty known as “forensic epidemiology,” as well as in graduate training curricula, continuing education programs, and conferences, with the recognition that it is an extension of widely accepted ethics guidelines for epidemiological research.

**Conclusion:**

We welcome feedback from the research community about ways to strengthen the Toolkit as it is applied to a broader assemblage of research studies and disciplines, contributing to its value as a living tool/instrument. The application of the Toolkit by Tsuda et al. exemplifies the usefulness of this framework for transparently evaluating, in a systematic way, epidemiological research, conclusions relating to causation, and policy decisions.

**Postscript:**

We note that our Toolkit has, most recently, inspired authors with discipline-specific expertise in the field of Conservation Biology to adapt it for use in the Biological Sciences.

## Background

Tsuda et al. [1] recently applied our Toolkit for detecting misused epidemiological methods [2] that serves to organize and formalize the transparent evaluation of research papers or reports. Our focus in this comment is not on the specialized content area of Tsuda et al., but rather on the recommendations made by them to improve and manage the Toolkit. In addition, the potential value of the Toolkit in the training of professionals in our field, as well in the sub-specialty of forensic epidemiology, is emphasized.

## Response to recommendations to improve the Toolkit

We welcome the suggestion by Tsuda et al. [1] that the items in Part A (i.e., method-related misuses) of the Toolkit [2] be rearranged in the order of how a study is designed, conducted, analyzed, and reported. This could help other potential users of the Toolkit navigate through all of the items. Most items in the Toolkit apply to epidemiological studies individually, or to a collection of topic-related studies that constitute the body of evidence, including review articles, meta-analyses, and policy documents. The suggestion to expand the Toolkit to other related health disciplines, such as mechanistic studies, requires methodological expertise across a variety of disciplines. The Toolkit could indeed be adapted to transparently evaluate studies beyond epidemiology, paving the way to multi- and inter-disciplinary collaborations leading to even more useful toolkits.

Additional Part A items suggested by Tsuda et al., are, on consideration, adequately covered by existing items in the Toolkit and, as noted, they do sometimes overlap. We did not consider intention, that is whether a problem with a study is “deliberate” or “intentional,” because our aim is to help reviewers identify flaws in a body of evidence, regardless of how these flaws were created. One of us (L.A.B.) has grappled with a similar issue regarding the identification of problematic studies in systematic reviews [3]. There, she and her coauthors found that intention does not really matter when the objective is to identify untrustworthy studies, but that it has legal implications. In our Toolkit, we are interested in identifying misused (i.e., bad) studies.

## Caution against using a simple tally to suggest the extent of misuse

We caution against the simple tallying of overall Toolkit items (n = 33) or reporting the proportion of items met for comparing the extent of misuse across studies. In so doing, this suggests a score based on the Toolkit per se. The purpose of the Toolkit remains one of assisting educators, reviewers, researchers, and policymakers to identify how epidemiological studies can be flawed and misinterpreted. It was not designed to produce an overall, cumulative score. Since none of the items are weighted, an overall score would be misleading in making comparisons. At minimum, scores across each of the three dimensions of the Toolkit (i.e., Part A, *Methods / Techniques* [n = 18]; Part B, *Arguments* [n = 8]; and Part C, *Tactics* [n = 7]) could be more meaningful. We suggest, instead, however, a visual representation of how a study meets the Toolkit items, such as the heat maps used to present risk of bias criteria for studies included in Cochrane reviews [4]. Akin to risk of bias assessment, it might be appropriate to have more than one reviewer independently assessing the studies; in fact, at least two evaluators are normally required for risk of bias assessment in Cochrane reviews [4]. Furthermore, it would be interesting to explore the level of consensus and inter-rater agreement when the same studies are evaluated by multiple reviewers using the Toolkit.

## Reinforcing the role of ethics in professionalism

We note that the principles delineated in the Toolkit are complementary to long-standing and widely accepted ethics guidelines for epidemiologists and biomedical researchers [5–7]. These guidelines were developed to raise awareness of, among others, undeclared conflicting interests, transparency, erroneous assumptions, and unethical behavior carried out by our own colleagues in the field of epidemiology, emanating from public health agencies, scientific journals, international expert panels, and academia. The guidelines are intended to prevent such breaches. We can only re-emphasize the critical importance of incorporating into graduate training curricula, continuing education programs, and research conferences such topics as they relate to professional ethics and research integrity. Every opportunity to broaden discussion, and to increase both awareness and understanding of ethics writ large in the health professions should be taken, especially by mentors. This applies not only to epidemiology, but across all specialty and sub-specialty areas in the health sciences.

## Enhancing the evaluative and forensic utility of the Toolkit

We look forward to learning of additional applications of our Toolkit and would welcome further suggestions for its improvement. Its wider application globally would likely help with its refinement and utility.

The Toolkit, in our view, has potential value to the branch of evaluative public health science currently defined as “forensic epidemiology” [8]. We recommend that professionals in our field receive training, incorporating our Toolkit, allowing for a systematic approach in transparently deconstructing the way that the effect of exposures on health has been, and continues to be, misused in relation to health effects.

## The Toolkit as a living tool/instrument

We believe that a system by which examples of misuse are accumulated should be created. Examples of misuse could serve as a reference for assessing the utility of the Toolkit in protecting the public’s health and for its further enhancement. This task could be undertaken by an established public health agency or a professional organization whose members are not entirely made up of volunteers. In this way, the Toolkit would become a living document; its periodic updates could be made accessible on a website. We propose that the *International Society for Environmental Epidemiology* (ISEE) be asked to consider taking on this task.

## Conclusion

As awareness of the utility of toolkits increases, their application to various sub-specialty areas within the field of epidemiology will further help to identify their respective strengths and weaknesses, paving the way to more refined and useful toolkits. With ever-expanding research outputs globally, toolkits could well become an effective aid in the evaluation process, facilitating transparency in critiques of published studies.

## Postscript

A year after publication of our Toolkit article [2], not only do we see its application by Tsuda et al. [1] in the field of Epidemiology, but now, a few weeks later in *Conservation Letters*, a journal of the Society for Conservation Biology, our Toolkit is seen to have inspired its authors to fashion it for use in the Biological Sciences [9]. Burgman et al. [9] have used our Toolkit [2] as a starting point and adapted the items to reflect issues of special relevance to Conservation Biology, focusing on issues that are relevant in conservation and environmental science. We are gratified to see, as noted above, awareness of the utility of toolkits increasing, not only in the field of Epidemiology, but now too its utility to a broader assemblage of research studies and disciplines, the latest being the field of Conservation Biology, a field where rational evidence for preventing harms on an ecological and global scale is so critical. This wider application of our Toolkit [2] globally can only help with its refinement and utility in serving the public interest.

## Data Availability

This declaration is not applicable.
